# Role of Resilience in Predicting and Empathy Dimensions in Dental Students: A Cross-Sectional Design

**DOI:** 10.3390/bs15060769

**Published:** 2025-06-03

**Authors:** Victor P. Díaz-Narváez, Nuvia Estrada-Méndez, Jennifer Aldana Salguero, Brenda Alfaro Ortiz, Lindsey Vilca Quiro, Alejandro Reyes-Reyes, Carolina Alzugaray Ponce

**Affiliations:** 1 Research Department, Universidad Andres Bello, Santiago 8320000, Chile; 2 Research Department, Universidad Evangélica de El Salvador, San Salvador 1789, El Salvador; nuvia.estrada@uees.edu.sv (N.E.-M.); jennifer.aldana@uees.edu.sv (J.A.S.); brenda.alfaro@uees.edu.sv (B.A.O.); 3 Research Department, Universidad Señor de Sipán, Chiclayo 14002, Peru; vilcaquiro@uss.edu.pe; 4 Department of Psychology, Faculty of Social Sciences and Communications, Universidad Santo Tomás, Concepción 3360001, Chile; areyesr@santotomas.cl (A.R.-R.); carolinaalzugaray@santotomas.cl (C.A.P.)

**Keywords:** resilience, empathy, dental students

## Abstract

Ecological empathy is a complex and multifaceted attribute, a highly relevant human factor in health care. This study aimed to determine the association between individual resilience and empathy in dental students and to assess whether resilience predicted empathy and its dimensions. The study employed a quantitative and descriptive cross-sectional design. The sample, consisting of 397 students from the first to the seventh year of dental surgery, was measured on empathy and resilience. Multiple regression analysis, confirmatory factor analysis, invariance analysis, and methods were employed to ensure the reliability of our measurements. It was established that the measurements of resilience and empathy were valid and reliable in the studied population. Ecological resilience increased the levels of the perspective adoption dimension, and adaptive resilience raised the compassionate threshold. The engineering resilience dimension was not associated with any of the empathy dimensions. While resilience may not fully explain empathy variability, it remains a personal attribute related to empathy. Further investigation of the role of resilience in empathy and its components is required since improving healthcare professionals’ empathy has positive implications for the quality of care and patient satisfaction.

## 1. Introduction

Medical empathy is considered an attribute that enables the comprehension of patients’ thoughts and feelings and allows the treating physician to respond appropriately from an emotional standpoint (Adam et al., 2022). As a result, empathy is a construct structured by two components: the cognitive one, which provides the treating medical professional with an understanding of the patient’s experiences and expectations regarding their health situation (Ulloque et al., 2019), and the emotional component, which allows the medical professional to understand and feel the patient’s suffering (Tehranineshat et al., 2019; Thompson et al., 2019). Therefore, it is essential to consider empathy as a complex and dynamic system since its components are dialectically interconnected (Altuna, 2018). As a consequence of those above, an imbalance in this equilibrium results in a decrease in empathetic capacity and, in the worst case, severe psychopathology when one of these components has a critical flaw (Blair, 2018; Kerr-Gaffney et al., 2019). The cognitive component has two dimensions, perspective adoption (PA) and “walking in the patient’s shoes” (WIPS), while the emotional component consists of one dimension, compassionate care (CC). The complexity of the empathic development process in a human being is not limited to the interaction between the components or their dimensions. Indeed, two factors are involved in developing empathy in humans: phylogeny and ontogeny (Decety & Svetlova, 2012). The former is the product of biological evolution and the changes it entails, including those in the brain, which were (and still are) slow processes. This process serves to accumulate positive adaptive changes in the form of genetic information and, over time, allows the formation of the limbic system of the brain, which is responsible for a person’s emotional processes and is subsequently involved in the development of the frontal lobe, which can be associated with the development of cognitive capacity in the human being (Barrera-Gil et al., 2018). The latter (ontogeny) is composed of all the factors that can influence a person throughout their existence and starts since birth or even before. Therefore, it is necessary to study factors that may be “suspected” of contributing to or hindering the normal development of the brain structures that support empathy components, the interconnections (neural networks) between these structures, and the functioning of said structures and interconnections (Couette et al., 2022). For this reason, numerous factors in the ontogeny process can influence empathy in early childhood, adolescence, or young adulthood. These factors are also present in the formation process of students in various healthcare fields. Some of these factors may be: child abuse (Berzenski & Yates, 2022), family violence (Ramos et al., 2021), bullying (Garandeau et al., 2022), burnout (Lopes & Nihei, 2020), academic stress (Banerjee et al., 2019), personality (Dávila-Pontón et al., 2020a), family functioning (Dávila-Pontón et al., 2020b), culture (Ullrich, 2022), prosocial behaviors (Suazo et al., 2020), and resilience (Spilg et al., 2022), among many others. Some of these factors can critically influence empathic development (Allen et al., 2021; Hartman et al., 2019; Metcalf et al., 2021), and their outcomes can determine the presence of a decrease in empathy or be associated with psychopathologies. Given these multiple implications, empathy studying empathy determinants is highly relevant.

There are several definitions of the concept of “resilience”. This variability in this construct’s definition resides in its theoretical foundations and associated characteristics (Bonanno, 2021; Tang et al., 2022; Young et al., 2019). However, all these theories agree that resilience is complex and determined by several variables or traits attributed to it, which are not necessarily wholly independent and can interact with each other.

There are a series of risk factors generally associated with resilience, such as experiencing depression, anxiety, or abuse, as well as protective factors like positive affect, self-esteem, optimism, or self-efficacy, among many others (Hunsu et al., 2023). Regarding risks, there seems to be agreement among authors on the association between exposure to significant risk and positive evolution in terms of psychosocial well-being despite the threat being faced (Yule et al., 2019), considering as “significant risk” a situation that appears to have no solution and may lead to dysfunctional adaptation and psychological distress (Yule et al., 2019). In this context, resilient individuals can adapt and face the negative experiences that may occur in their lives, thanks to the ability to generate a “positive evolution” of their life project despite adverse conditions that may arise in their environment. They acquire the ability to use personal, family, relational, and existential resources properly to cope with suffering and psychic vulnerability (Dulin et al., 2018).

However, the concept of resilience is used in many ways depending on the field of application and the theoretical-practical basis. This situation prevents obtaining a common definition of the concept. It makes it difficult to compare the results of different research (Moya & Goenechea, 2022), with the implication that the absence of a common definition complicates its objective measurement (Sisto et al., 2019).

The absence of a single theory of individual resilience in psychology makes its operationalization challenging. So far, there are two approaches: the buffering hypothesis and the trait approach (Johnson et al., 2011; Khalil et al., 2022). The buffering approach measures resilience on a binary scale, having or not having a specific characteristic (Heritage et al., 2019), whereas the trait approach examines how individuals cope with events they perceive as negative and takes into account their capacity for recovery (Maltby et al., 2015). Based on Holling’s approach (Holling, 2006), which combines the systems theory and ecology to describe resilience in various ecological systems, three subsystems are derived to explain resilience: engineering resilience, ecological resilience, and adaptive capacity. Engineering resilience is the ability to return to or recover equilibrium after disturbance (Hoffman & Hancock, 2017). Ecological resilience is the capacity of a system to absorb or resist a disturbance before realigning the key fundamental critical mechanisms of the system and maintaining its stable state in terms of function, purpose, structure, or identity (Maltby et al., 2015; Timpane-Padgham et al., 2017). Adaptive capacity is the ability of an ecosystem to manage and accommodate change and to adapt (Faye et al., 2018; Maltby et al., 2015; Palacio et al., 2020).

In the psychological literature, engineering resilience is an individual’s capacity to recover or “return” to their original state after challenging experiences (Howell & Miller-Graff, 2014). Ecological resilience has been recognized in the psychological literature as the ability to be robust; demonstrate confidence in one’s strengths and abilities; and be stoic, resourceful, and determined as one navigates through life (Golubovich et al., 2014; Vaughn & DeJonckheere, 2021). Adaptive capacity has been recognized in the psychological literature as the ability to adapt to adverse circumstances, be flexible when facing them, and have the capacity to modify said circumstances and respond effectively to disruptions (Connor & Davidson, 2003).

Empathy and resilience, along with other factors, have been studied to explain various behaviors in healthcare professionals and students of healthcare sciences (Chaukos et al., 2017; Elam & Taku, 2022; Foster et al., 2018; Rafaqat et al., 2022; Sturzu et al., 2019; Velayudhan, 2021). In this regard, a positive effect of resilience and self-efficacy was observed on compassion fatigue in nurses (Zhang et al., 2022). Compassion fatigue is one of the elements involved in the process of empathic erosion. The cited study demonstrated resilience by raising the nurses’ compassion threshold. It has also been observed that resilience acts as a mediator between empathy and work commitment (Cao & Chen, 2020).

In this sense, it has been demonstrated that dental students are subjected to the action of various factors that can cause a decrease in empathy toward the patient. These factors include stress (Nitschke & Bartz, 2023), depression (Rajput et al., 2020), burn-out (MacAulay et al., 2023), personal well-being (Paloniemi et al., 2021), and academic performance and anxiety (Ahmad et al., 2022), among other factors. The aforementioned factors could be modulated by resilience by eliminating or attenuating its negative effect on the cognitive and emotional components of empathy and reducing the risk of the presence of empathic erosion in dental students in particular and of the health sciences student, in general (Díaz-Narváez et al., 2020). In this sense, several authors have presented information that leads to considering that resilience could be an independent variable of empathy (Findyartini et al., 2021; Halimi et al., 2023; Morice-Ramat et al., 2018; Ord et al., 2020; Rafaqat et al., 2022; Stern et al., 2023; Waddimba et al., 2021; Wu et al., 2022). Additionally, it is necessary to consider the proven existence of variability in the distribution of empathy in dental students in Latin America (Díaz-Narváez et al., 2021), which implies that the main question to be answered is not whether there is an association between the two variables under study but how these variables are associated explicitly in a population of students. 

However, studies on resilience as a predictor of empathy and its dimensions have been relatively scarce. Specifically, studies on resilience as a predictor of empathy in dental students from Latin America and other continents are almost non-existent. Furthermore, studies of this type in Latin America must be carried out with particular care due to the significant variability in the distribution of empathy among students within the same specialty and across different specialties within and among countries of this continent. Empathy is a desirable competency for healthcare professionals, and it enhances the quality of patient care in a few ways (Cruz, 2020). Determining the associated factors and predictors of empathy, such as resilience, could improve empathic capacity by strengthening resilience. All these variables, taken together, can improve the clinical practice of dentists and the stress experienced by dental students and general and pediatric dentists (Tokgöz Kaplan, 2024).

Since empathy is an essential competency in the health care setting and resilience could influence its development, it is necessary to study the relationship between these variables. This study sought to investigate the psychometric properties of empathy and resilience measures in dental students and how resilience was associated with predicting empathy levels in this population. We set out with two consecutive objectives. The first was to establish the psychometric properties of empathy and resilience measures, including sex invariance. The second objective was to analyze the association between empathy and resilience to predict empathy values as a function of resilience in a population of dental students.

## 2. Methods

### 2.1. Design

A cross-sectional study was conducted.

### 2.2. Participants

The sample consisted of 397 dental surgery students from the first to the seventh year in 2022, distributed as 73.8% (*n* = 293) females and 26.2% (*n* = 104) males, with ages ranging from 18 to 33 years (*M* = 21.49, *SD* = 2.79). The participants were selected through convenience sampling from a population of 462 students of the Faculty of Dentistry “Evangélica University of El Salvador”, with 14.07% of the population of students who chose not to participate or were absent from classes during the data collection period.

### 2.3. Study Measures

The Jefferson scale of empathy (JSE-HPS) (Hojat et al., 2002b) consisted of 20 items that measured the levels of empathy with patients in health science students of any specialty. The items were rated on a 7-point response scale, ranging from 1 (strongly disagree) to 7 (strongly agree). The scale measured three dimensions: compassionate care (CC, items 1, 7, 8, 11, 12, 14, 18, and 19), perspective adoption (PA, items 2, 4, 5, 9, 10, 13, 15, 16, 17, and 20), and “walking in the patient’s shoes” (WIPS, items 3 and 6). The scale demonstrated adequate internal consistency (α = 0.78–0.92) and appropriate correlations with other psychological variables.

The resilience trait scale (EEA, Maltby et al., 2015) assessed three facets of resilience: engineering, ecological, and adaptive resilience. It had a 12-item Likert-type format, with five response levels per item, from “strongly disagree” (1) to “strongly agree” (5). The FSS demonstrated adequate internal and test–retest reliability, a cross-culturally stable factor structure, convergent and construct validity in terms of associations with personality, and a positive contribution to clinical and non-clinical psychological health states (Maltby et al., 2015, 2016).

### 2.4. Procedures

All students who agreed to participate signed an informed consent form before completing the instruments, ensuring their autonomy, voluntariness, and the confidentiality of information. All students were subjected to the same pedagogical strategy, emphasizing the teaching of human values.

Data collection for both study periods took place in September 2022. The instruments were administered in groups, in pen and paper format, during the students’ regular class schedule. Data were collected by professors from the Faculty of Dentistry who were not involved in this research and had received the necessary training to administer the instruments correctly, address student questions, and ensure the proper reception of responses. The following were considered inclusion criteria: voluntarily agreeing to respond to the instrument, having regular student status (basic, pre-clinical, or clinical), and being present during the instrument’s administration. All the evaluated students belonged to the Faculty of Dentistry of the Evangelical University of El Salvador (El Salvador).

The study adhered to the ethical principles of the Declaration of Helsinki (2013). The Institutional Ethics Committee of Andrés Bello University, Record 020-2022, approved the research project and informed consent. All sociodemographic and personal data and the responses from the administered instruments were considered confidential.

### 2.5. Data Analysis

To portray empathy and resilience, a series of descriptive statistics were calculated for each of the items and dimensions of the instruments. Cronbach’s alpha (α) and McDonald’s omega (ω) were used to assess the measure’s reliability. Values of α > 0.70 were considered to indicate “good” reliability. To establish differences by sex, the Student’s *t*-test was used for independent samples.

A confirmatory factor analysis (CFA) was conducted using the maximum likelihood estimation method to determine the validity of the resilience and empathy models. To assess the goodness of fit, the relative chi-square (CMIN/DF) was estimated along with the chi-square and degrees of freedom, the goodness of fit index (GFI), the comparative fit index (CFI), the non-normed fit index (NNFI), the root mean square error of approximation (RMSEA), and the standardized root mean square residual (SRMR), following the recommendations of Hoyle (1995), Hu and Bentler (1999), and Kline (2005). Acceptable fit was indicated by a CMIN/DF of less than 2 or 3, GFI and CFI of at least 0.90, RMSEA index between 0.05 and 0.08, and SRMR less than 0.08.

Factorial invariance was analyzed through a multigroup-analyzed model (Jöreskog, 1971b), using the Chi-square test (χ^2^) to assess the goodness of fit. However, since the Chi-square test was sensitive to the sample size, decreases in CFI of less than 0.01 (Δ ± 0.01) compared with the previous model were considered a more appropriate indicator of invariance (Cheung & Rensvold, 2002).

Empathy (E) and its dimensions (CC, PT, and WIPS) were considered dependent variables, while engineering resilience, ecological resilience, adaptive resilience, and age were treated as independent variables. The analysis was weighted by sex due to the imbalance in this variable in the sample. The values of multiple R, multiple R^2^, adjusted R^2^, the F-value from the analysis of variance, the Durbin–Watson statistic (DW), tolerance statistic, and variance inflation factor (VIF) were estimated. Descriptive statistics were calculated using SPSS 27, and the confirmatory factor analysis (CFA) was performed using AMOS 25 on the collected data based on the three-factor resilience model.

## 3. Results

Table 1 shows that all items had skewness (Sk) and kurtosis (Kurt) values within the expected limits (Sk < ±2; Kurt < ±7).

Table 2 presents the results for the mean and standard deviation levels of E, CC, PT, PA, and WIPS in the factors Y, C, and S, as well as combinations of these factors’ levels.

**Reliability:** The dimensions of the empathy scale had adequate reliability indices: PT or PA (α = 0.89; ω = 0.89), CC (α = 0.79; ω = 0.79), and WIPS (α = 0.68; ω = 0.68). Similarly, in the sample of females, all three dimensions had acceptable reliability indices: PA (α = 0.89; ω = 0.89), CC (α = 0.77; ω = 0.77), and WIPS (α = 0.64; ω = 0.64). In the sample of males, the three dimensions also presented adequate adjustment indices: PA (α = 0.89; ω = 0.89), CC (α = 0.83; ω = 0.83), and WIPS (α = 0.74; ω = 0.75). **Validity based on the internal structure:** It was evident that the original model of three related factors presented adequate adjustment indices (χ^2^ = 329.87; df = 167; *p* < 0.01; IFC = 0.92; TLI = 0.91; RMSEA = 0.056 [90% CI 0.047–0.064]; SRMR = 0.057). In addition, the factor weight of most of the items was adequate. It was also clear that the dimensions were related to each other. **Factorial Invariance:** In Table 3 it is observed that the factorial structure of the scale showed evidence of being strictly invariant for the groups of males and females in the sequence of proposed invariance models: metric invariance (ΔCFI = −0.001; ΔRMSEA = −0.002), scalar (ΔCFI = −0.010; ΔRMSEA = 0.002), and strict (ΔCFI = −0.003; ΔRMSEA = −0.001).

### 3.1. Resilience

Table 4 presents the descriptive statistics of the items and dimensions of resilience. The skewness (Sk) and kurtosis (Kurt) values of the items and the dimensions were within the range of +/−1, indicating a “very good” symmetry of a univariate normal distribution.

In addition, the Cronbach’s alpha coefficients for the scales (engineering resilience, α = 0.86; ecological resilience, α = 0.80; and adaptive resilience, α = 0.75), with α = 0.84 for the total scale, surpass the aforementioned internal reliability criterion of α > 0.70, which is considered “good”. Finally, differences in resilience were found based on sex, with females (*M* = 40.86, *SD* = 7.15) exhibiting lower resilience than males (*M* = 44.69, *SD* = 7.55) (t = 4.628, df = 395, *p* = 0.0001).

When establishing the factorial validity of the resilience scale through CFA, significant factor loadings were observed, ranging between λ = 0.538 and λ = 0.839 (Table 5), with goodness-of-fit statistics for the three-factor model that met the aforementioned criteria of adequate fit to the data, incorporating correlated errors for items 3 and 4 (χ^2^ = 123.968, df = 50, *p* < 0.001; χ^2^/df = 2.479; GFI = 0.952, CFI = 0.96, RMSEA = 0.061, 90% CI for RMSEA = 0.048–0.075, SRMR = 0.0445). When examining the goodness of fit of the model for both sexes, an adequate fit was observed for females, χ^2^ = 110.331, df = 50, *p* < 0.001; χ^2^/df = 2.207; GFI = 0.944, CFI = 0.954, RMSEA = 0.064, 90% CI for RMSEA = 0.048–0.081, SRMR = 0.0448, as well as for males, χ^2^ = 89.845, df = 50, *p* < 0.001; χ^2^/df = 1.797; GFI = 0.877, CFI = 0.92, RMSEA = 0.088, 90% CI for RMSEA = 0.058–0.117, SRMR = 0.0862.

### 3.2. Sex Invariance Indices of the Resilience Scale

A factorial invariance analysis was conducted, comparing females and males through a multigroup analysis, revealing a reasonably adequate fit of the model to the data: χ^2^ = 200.411, *p* < 0.0001, χ^2^/df = 2.004, SRMR = 0.047, GFI = 0.925, CFI = 0.945, RMSEA = 0.050 (90% CI = 0.040–0.061). After establishing the baseline models by sex and nested models based on the baseline model, no significant changes were observed in the chi-square value, with differences in CFI that were not relevant (ΔCFI < 0.01), which was less than 0.01, allowing the assumption of configural and metric invariance (Cheung & Rensvold, 2002) (Table 6).

Table 7 shows the results of the ANOVA F-value. All of them were significant or highly significant, except the dependent variable WIPS. This meant that the correlation coefficient values (R) of the corresponding regression equations were significant (different from zero). In all cases, the DW statistic was not significant and ranged between the values of 1.5 and 2.5, implying that there was no autocorrelation. The residuals were distributed independently of each other (Savin & White, 1977). Finally, the regression coefficient values and determination coefficients found for the significant variables showed that the degree of association fluctuated between 18.2% and 22.5%, and the variance explained by these variables fluctuated between 3.3% and 5.0%.

Table 8 shows the regression analysis results (weighted by sex). Ecological resilience was positively associated with empathy, CC was negatively associated with adaptive resilience, and WIPS was not associated with any of the independent variables. The associations described above were highly significant in all cases, with tolerance values greater than 0.1 and VIF values less than 10, indicating that the independent variables were not collinear. Age was not significant; therefore, in the studied sample, it did not influence empathy levels and dimensions.

## 4. Discussion

Our first objective was to establish the psychometric properties of the measures used, including sex invariance. The psychometric analyses conducted on measurements of resilience and empathy allowed us to demonstrate that the model of three underlying dimensions in each of them was met in the sample of the population of dental students. This finding was important because an a priori assumption should not be made that the model of a construct fit the collected data, even in samples taken from the same population in which this compliance had been observed before, especially if the context changed (Velickovic et al., 2020). Additionally, the invariance analysis allowed us to observe that the model of both constructs held for both sexes. This finding supported the reliability of sex comparisons (de Roover et al., 2022). Furthermore, the regression model complied with the assumptions for its robustness, validating the model, and therefore, the associations found could be considered reliable in the studied sample.

Empathy is a complex construct and has been the subject of extensive theoretical discussions without reaching a complete agreement (Eisenberg & Fabes, 1990; Eisenberg et al., 1991; Férriz et al., 2018; Machado & Calvetti, 2019). Nevertheless, there is agreement that medical empathy has both cognitive and emotional components (Hojat et al., 2002a). In the literature, it has been observed that there are some differences in the definition in the sense that some authors consider clinical empathy to be predominantly cognitive when it comes to patient care (Hojat et al., 2002a, 2020, 2023). However, other authors (Díaz-Narváez et al., 2022) suggest that empathy is a system composed of dimensions, and none of these dimensions is predominant over the others; instead, there is a dialectical relation between these components, and this relation is dynamic. As a system, empathy requires the active participation of its three dimensions. Therefore, one cannot be empathetic with the patient if the three dimensions do not operate in the process of intersubjectivity with the patient. A similar situation occurs with individual resilience, which is characterized by a lack of clarity regarding the exact meaning of this construct and its constituents (Alzugaray et al., 2018). The trait approach is characterized by the ability to combine systems theory and ecology to describe resilience. Therefore, attempting to study how a complex system, such as resilience, can modulate another complex system, such as empathy, is a difficult task that allows only approximate knowledge of the interactions between both systems. Indeed, resilience could be generally defined by a high degree of expectation, self-determination, flexibility, optimism, cognitive reappraisal, and active coping (Han & Nestler, 2017).

In this sense, it is known that dental students are exposed to exogenous disturbances that hinder their academic and clinical performance. Stress, anxiety, depression, academic pressure, workload, financial problems, year of study, exams, grades, and patient care are among these factors (Fonseca-Molina et al., 2018a, 2018b). The students’ response to this situation should positively face these disturbances (Atkinson et al., 2009). In this regard, the findings suggested that measures of resilience were related to adaptive traits (extroversion, agreeableness, openness, and conscientiousness) and adaptive coping traits (problem-focused coping, positive appraisals, and emotional regulation) (Gariépy et al., 2016; Alzugaray et al., 2020).

The results observed in this study showed that adaptive resilience was negatively associated with the empathy component CC. Therefore, we should expect that the levels of this dimension were somewhat deficient compared with the maximum score that could be achieved in this dimension (56 points), and this should be observed empirically. Therefore, if adaptive resilience is characterized by the ability to adapt well, adjust, be flexible, change, innovate, modify, and respond effectively to disturbances (Maltby et al., 2015), it means that the presence of these traits will lead to a decrease in the levels of the CC component (a decrease in the empathetic threshold). Indeed, the values of this dimension, according to cutoff points recently calculated for dental students in Latin America, can be characterized as high but with values lower than the 25th percentile.

The possible consequences of this finding could be explained by the potential presence of some degree of empathic fatigue and, consequently, empathic erosion in students, with the negative impact this has on the student–patient relationship. However, it is also possible to speculate that this finding could be explained by a conscious process of adaptation to the conditions of observing pain and an excellent response to this disturbance, primarily using cognitive empathy to find the necessary “mechanism” to maintain an objective perspective and make informed decisions, based on the recognition that emotional empathy is also necessary to establish an effective connection with patients. Therefore, what was described above may imply the ability to “apply” both cognitive and emotional empathy in different situations and to be aware of when it is appropriate to “utilize” each of them with a greater “intensity” in relation to the other in order to create a suitable empathetic balance that allows the regulation of the effect of the patient’s pain on the student’s empathic effectiveness and ensures appropriate patient care. This alternative can explain the coping success based on the idea that adaptive resilience produces an increase in the compassionate threshold and that this process possibly allows the student to positively adapt to disturbances through emotional regulation mechanisms without this regulation diminishing their compassionate capacity. In this case, resilience would contribute to finding a balance without affecting their ability to feel compassion. Certainly, a response to this inference should be studied in future research.

On the other hand, the positive association found between ecological resilience and the PA component of empathy can be understood in the sense that the traits contained in this resilience (the ability to be robust, self-confidence, confidence in one’s abilities, stoic attitude, resourcefulness, and determination, which are achieved through critical domains in one’s life) can be associated with the development of executive functions in a complex process. In this sense, cognitive development is additive-systemic throughout the ontogeny process: selectivity and control of cognitive processes, increased ability to create mental schemas, greater mental flexibility, increased use and complexity of memory-learning strategies, and improved organization and planning of cognitive and behavioral activity. Based on constant development of the attitude toward analyzing things in increasingly abstract ways, as well as using more complex psycholinguistic elements (Ganesan & Steinbeis, 2022; Shibaoka et al., 2023), the development of these cognitive aspects, in turn, favors the specific cognitive aspects of the PA component of empathy. Indeed, Decety and Jackson (2004) stated that cognitive flexibility is required to adopt the perspective of others, and the traits of ecological resilience could, in some way, positively influence the ability to develop the necessary cognitive flexibility to develop the PA dimension in empathy positively. Indeed, this inference needs to be subjected to further research that can determine the “intersection zones” between the traits of ecological resilience, the ontogenetic cognitive development of executive functions, and the ability to develop patient perspective adoption, which is ultimately the intellectual understanding of their condition.

The lack of association between resilience in general and the WIPS dimension in the present study is difficult to explain. On the one hand, it is known that the WIPS dimension provides the ability to understand the meaning of the other person’s thoughts without losing the objectivity of that meaning. Thus, the presence of a negative association between adaptive resilience, which raises the threshold of compassion (possibly without losing this capacity); the positive association of ecological resilience with respect to the PA dimension; and possibly the absence of highly intense events in the process of providing patient care that would motivate an alteration of the empathic balance could be the necessary and sufficient conditions for each type of resilience to influence, in some way, the levels of WIPS. Regarding the resilience construct, it has been observed that the engineering dimension, when measured in adolescents, may be less significant in relation to the other dimensions (Alzugaray et al., 2020), which could diminish the overall effect of resilience.

Finally, the role of certain sociodemographic variables, such as age, sex, education, and income, which are associated with resilience and could be confounding factors, must be further investigated (Ueno et al., 2021). Recent studies suggest that females may be less resilient (Afshari et al., 2021; Singh et al., 2019) and that resilience increases with age (Afshari et al., 2021; Staneva et al., 2022).

Among the study’s limitations, we can note that the results of the associations found cannot be generalized a priori to all dental students in El Salvador and Latin America, due to the significant variability in empathy and its dimensions by sex, country, and different forms that empathy levels and its dimensions take throughout courses (empathic decline).

## 5. Conclusions

There is an existing association between resilience and empathy. However, the associations found make it possible to distinguish and specify that some dimensions of resilience are associated with some dimensions of empathy. Specifically, ecological resilience increases the levels of the perspective adoption dimension (intellectual understanding of the patient’s condition), and adaptive resilience raises the compassionate threshold (the ability to understand and comprehend the patient’s pain, whether psychological, physical, or both). Engineering resilience is not associated with any of the dimensions of empathy. The findings of this study can be used to help cultivate empathy and resilience in future healthcare professionals. We propose the need to conduct large-scale studies in different dental schools and colleges in Latin America to find more empirical evidence about the prediction of empathy based on resilience, which would allow for a degree of theoretical generalization regarding how resilience can effectively eliminate the negative effects of empathic inhibition on students. The results of these research actions can form the basis for serious and responsible interventions for robust, resilient, and empathetic training.

## Figures and Tables

**Table 1:** Descriptive analysis of the items of the Jefferson scale of empathy (HPS version).

Items	M	SD	Sk	Kurt
1. The understanding that health professionals have about the feelings of their patients and their families does not influence the treatment outcome.	4.28	2.04	−0.13	−1.23
2. Patients feel better when the health professional understands their feelings.	5.90	1.38	−1.37	−0.53
3. It is difficult for health professionals to see things from the perspective of his patients.	3.80	1.61	0.15	−0.53
4. Understanding body language is as important as verbal communication in the relationship between a health professional and his patients.	5.95	1.34	−1.35	1.57
5. A health professional’s sense of humour contributes to better clinical outcomes.	5.49	1.56	−0.88	0.01
6. Because people are different, it is difficult to see things from the patient’s perspective.	3.46	1.57	0.34	−0.22
7. Paying attention to the emotions of patients is not important during the anamnesis.	4.95	2.15	−0.64	−1.05
8. Considering the personal experiences of patients does not influence the treatment outcomes.	4.33	2.03	−0.14	−1.21
9. Health professionals should try to put themselves in their patients’ shoes when caring for them.	5.48	1.49	−0.81	−0.05
10. Patients value when health professionals show understanding of their feelings, which is therapeutic in itself.	5.75	1.41	−1.16	0.94
11. Patients’ diseases can be cured only by specific treatments; therefore, the emotional bond between health professionals and their patients has no significant influence on the outcome of specific treatments.	4.17	1.89	0.05	−1.08
12. Asking patients about what is happening in their personal lives does not help in understanding their physical problems.	4.17	2.03	−0.04	−1.24
13. Health professionals should try to understand what is going on in the minds of their patients by paying attention to nonverbal aspects and body language.	5.33	1.56	−0.77	−0.13
14. I think emotions have no relevance in the treatment of medical illness.	4.58	1.99	−0.32	−1.13
15. Empathy is a therapeutic skill without which the success of the health professional is limited.	5.90	1.38	−1.21	0.79
16. The health professional’s understanding of the emotional state of his patients, as well as that of their families, is an important component in the relationship between the health professional and his patients.	5.61	1.39	−0.89	0.27
17. Health professionals should try to think like their patients in order to provide better care.	5.44	1.49	−0.69	−0.18
18. Health professionals should not allow themselves to be influenced by strong personal ties with their patients and their families.	3.24	1.81	0.47	−0.68
19. I don’t like reading non-medical literature or the arts.	4.62	1.95	−0.28	−1.11
20. I believe empathy is an important therapeutic factor in the treatment of patients.	6.07	1.43	−1.71	2.48

Note: M, arithmetic mean; SD, standard deviation; Sk, asymmetry; Kurt, kurtosis.

**Table 2:** Results of estimating the mean and standard deviation of empathy, compassionate care, perspective adoption, and walking in the patient’s shoes in the factors evaluated for academic year, educational level, sex, and their combinations.

		Female			Male			Total		
	E. Level	n	M	SD	n	M	SD	n	M	SD
Empathy	First	69	97.04	15.25	26	97.19	18.21	95	97.08	16.01
	Second	37	101.19	16.93	11	91.09	10.38	48	98.88	16.15
	Third	40	101.15	18.68	12	90.50	12.05	52	98.69	17.85
	Fourth	57	103.11	15.07	13	94.08	15.19	70	101.43	15.40
	Fifth	28	107.14	14.63	8	105.38	14.74	36	106.75	14.46
	Sixth	37	93.11	11.89	19	89.16	15.16	56	91.77	13.09
	Seventh	25	96.92	17.15	15	100.67	17.69	40	98.33	17.23
	Total	293	99.76	16.03	104	95.05	15.98	397	98.53	16.13
CC	First	69	33.64	9.62	26	34.50	11.39	95	33.87	10.08
	Second	37	35.14	10.97	11	31.45	6.09	48	34.29	10.13
	Third	40	36.38	10.22	12	29.42	5.44	52	34.77	9.75
	Fourth	57	35.79	9.58	13	31.92	12.34	70	35.07	10.16
	Fifth	28	37.61	9.57	8	36.63	9.33	36	37.39	9.39
	Sixth	37	33.22	7.88	19	29.53	10.24	56	31.96	8.83
	Seventh	25	35.96	11.36	15	31.73	14.20	40	34.38	12.49
	Total	293	35.14	9.83	104	32.13	10.62	397	34.35	10.12
PA	First	69	55.65	9.82	26	55.54	10.01	95	55.62	9.81
	Second	37	58.35	11.31	11	53.73	9.737	48	57.29	11.05
	Third	40	56.92	12.40	12	54.83	8.53	52	56.44	11.58
	Fourth	57	60.19	8.06	13	55.85	9.88	70	59.39	8.52
	Fifth	28	61.75	7.46	8	62.50	7.50	36	61.92	7.37
	Sixth	37	52.68	10.08	19	52.21	10.54	56	52.52	10.14
	Seventh	25	54.32	12.60	15	63.00	5.44	40	57.58	11.24
	Total	293	57.14	10.47	104	56.31	9.68	397	56.92	10.27
WIPS	First	69	7.75	2.82	26	7.15	2.88	95	7.59	2.83
	Second	37	7.70	2.71	11	5.91	2.51	48	7.29	2.74
	Third	40	7.85	2.89	12	6.25	3.36	52	7.48	3.05
	Fourth	57	7.12	2.68	13	6.31	2.32	70	6.97	2.62
	Fifth	28	7.79	2.28	8	6.25	3.01	36	7.44	2.50
	Sixth	37	7.22	2.71	19	7.42	2.74	56	7.29	2.70
	Seventh	25	6.64	2.33	15	5.93	3.35	40	6.37	2.73
	Total	293	7.48	2.69	104	6.62	2.87	397	7.25	2.76

Note: n = the sample size observed for each factor and levels of the factors examined. SD = standard deviation.

**Table 3:** Sex invariance indices of the empathy scale.

According to Sex	χ^2^	df	*p*	SRMR	TLI	CFI	RMSEA [CI 90%]	Δχ^2^	Δdf	*p*	ΔCFI	ΔRMSEA
Females	298.69	167	<0.01	0.063	0.90	0.91	0.058 [0.048–0.069]	–	–	–	–	–
Males	255.86	167	<0.01	0.079	0.87	0.88	0.073 [0.055–0.090]	–	–	–	–	–
Configural	558.69	334	<0.01	0.067	0.89	0.90	0.063 [0.053–0.072]	–	–	–	–	–
Metric	580.37	351	<0.01	0.070	0.89	0.90	0.061 [0.052–0.070]	20.26	17	0.261	−0.001	−0.002
Scalar	621.15	368	<0.01	0.072	0.89	0.89	0.063 [0.054–0.071]	42.93	17	0.000	−0.010	0.002
Strict	642.07	388	<0.01	0.075	0.89	0.89	0.062 [0.053–0.070]	24.36	20	0.227	−0.003	−0.001

Note: χ^2^, Chi square; df, degrees of freedom; SRMR, standardized root mean square residual; TLI, Tucker–Lewis index; CFI, comparative fit index; RMSEA, root mean square error of approximation; Δχ^2^, differences in Chi square; Δdf, differences in degrees of freedom; ΔRMSEA, change in root mean square error of approximation; ΔCFI, change in comparative fix index.

**Table 4:** Descriptive statistics of individual resilience and its dimensions (n = 397).

Items	Min	Max	M	SD	Sk	Kurt
1. I can easily recover from stressful events.	1	5	3.40	1.05	−0.263	−0.482
2. I quickly bounce back from a tough moment.	1	5	3.21	1.05	−0.056	−0.712
3. I can quickly return to my normal state after facing problems in my life.	1	5	3.28	1.04	−0.108	−0.546
4. I can easily return to my normal state after facing difficult or hard experiences.	1	5	3.18	1.09	−0.035	−0.713
5. I always give my all, no matter the outcome.	1	5	4.08	0.83	−0.868	0.874
6. I stay strong, no matter what problems arise.	1	5	3.71	0.93	−0.533	0.218
7. Even when there are problems, I am able function properly to accomplish my goals.	1	5	3.69	0.97	−0.628	0.133
8. No matter what happens, I always find a way to get things done.	1	5	3.90	0.83	−0.567	0.338
9. I enjoy it when life changes.	1	5	3.53	1.06	−0.417	−0.286
10. I like facing unpredictable situations.	1	5	3.06	1.12	−0.055	−0.589
11. Uncertain situations pique my interest.	1	5	3.26	1.11	−0.359	−0.495
12. I enjoy it when there are changes in my routine.	1	5	3.57	1.14	−0.587	−0.301
Engineering dimension	4	20	13.07	3.55	−0.025	−0.354
Ecological dimension	4	20	15.38	2.82	−0.519	0.591
Adaptive dimension	4	20	13.42	3.34	−0.256	−0.056
Resilience	12	60	41.86	7.44	−0.070	0.285

Note: M, mean; SD, standard deviation; Sk, skewness; Kurt, kurtosis.

**Table 5:** Factorial loadings and reliability of the resilience model.

Resilience	Variables	Factor Loadings	A	ω
Engineering dimension	P1	0.731	0.859	0.88
	P2	0.839		
	P3	0.774		
	P4	0.678		
Ecological dimension	P5	0.556	0.800	0.82
	P6	0.746		
	P7	0.779		
	P8	0.751		
Adaptive dimension	P9	0.701	0.746	0.75
	P10	0.701		
	P11	0.538		
	P12	0.670		
Resilience			0.843	0.87

**Table 6:** Goodness-of-fit of the multigroup confirmatory factor model by sex in successive nested models.

Model	χ^2^	df	*p*	Δχ^2^	Δdf	*p*	CFI	ΔCFI
Invariance by sex								
Configural	200.411	100	0.000	--	--	--	0.945	--
Metric	204.923	109	0.000	4.512	9	0.875	0.947	0.002
Scalar	208.336	115	0.000	3.413	6	0.756	0.949	0.002

**Table 7:** Results of the variance analysis in the regression analysis of empathy and its dimensions in relation to resilience and its dimensions.

Dependent Variable	F	*p*	DW	R	R^2^
E	3.549	0.007	1.676	0.187	0.035
CC	3.347	0.01	1.646	0.182	0.033
PA	5.208	0.0005	1.684	0.225	0.050
WIPS	1.253	0.288	1.913	0.112	0.013

Note: E, empathy; CC, compassionate care; PA, perspective adoption; WIPS, “walking in the patient’s shoes”; DW, Durbin–Watson statistic and variance inflation factor.

**Table 8:** Results of the multiple linear regression (weighted by sex) to determine the association between resilience and empathy.

	Unstandardized Coefficients			T	*p*	CI 95% β		Collinearity Statistics	
	β	EE	β_est._			LI	LS	Tol.	VIF
**Empathy**									
Engineering resilience	−0.438	0.260	−0.095	−1.682	0.093	−0.950	0.074	0.773	1.293
**Ecological resilience**	**1.170**	**0.325**	**0.206**	**3.596**	**0.0005**	**0.530**	**1.810**	**0.747**	**1.338**
Adaptive resilience	−0.473	0.262	−0.097	−1.805	0.072	−0.989	0.042	0.846	1.181
Age (years)	0.050	0.294	0.009	0.172	0.864	−0.527	0.628	0.997	1.003
**CC**									
Engineering resilience	−0.270	0.162	−0.094	−1.668	0.096	−0.589	0.048	0.773	1.293
Ecological resilience	0.311	0.202	0.088	1.538	0.125	−0.087	0.709	0.747	1.338
**Adaptive resilience**	**−0.429**	**0.163**	**−0.142**	**−2.627**	**0.009**	**−0.750**	**−0.108**	**0.846**	**1.181**
Age (years)	0.263	0.183	0.071	1.437	0.152	−0.097	0.622	0.997	1.003
**PA**									
Engineering resilience	−0.226	0.166	−0.076	−1.359	0.175	−0.553	0.101	0.773	1.293
**Ecological resilience**	**0.899**	**0.208**	**0.247**	**4.331**	**0.0004**	**0.491**	**1.307**	**0.747**	**1.338**
Adaptive resilience	−0.014	0.167	−0.005	−0.086	0.932	−0.343	0.315	0.846	1.181
Age (years)	−0.123	0.188	−0.032	−0.656	0.512	−0.492	0.246	0.997	1.003
**WIPS**									
Engineering resilience	0.058	0.045	0.074	1.295	0.196	−0.030	0.146	0.773	1.293
Ecological resilience	−0.040	0.056	−0.042	−0.723	0.470	−0.150	0.069	0.747	1.338
Adaptive resilience	−0.030	0.045	−0.037	−0.677	0.499	−0.119	0.058	0.846	1.181
Age (years)	−0.089	0.050	−0.089	−1.771	0.077	−0.188	0.010	0.997	1.003

Note: βest., standardized β coefficient; Tol., tolerance.

## Data Availability

The original contributions presented in this study are included in the article. Further inquiries can be directed to the corresponding author.

## References

[B1-behavsci-15-00769] Adam Z., Klimeš J., Boleloucký Z., Pour L., Adamová Z., Tomíška M., Marečková H. (2022). Patients benefi to from physicians empathy and results of including of empathy development into medical training. Klinicka Onkologie.

[B2-behavsci-15-00769] Afshari D., Nourollahi-darabad M., Chinisaz N. (2021). Demographic predictors of resilience among nurses during the COVID-19 pandemic. Work.

[B3-behavsci-15-00769] Ahmad P., Chaudhary F. A., Asif J. A., AlSagob E. I., Alkahtany M. F., Almadi K. H., AlMubarak A., Abduljabbar T., Vohra F. (2022). Risk analysis of factors in clinical anxiety among undergraduate and postgraduate students in dentistry. Work.

[B4-behavsci-15-00769] Allen A. B., Robertson E., Patin G. A. (2021). Improving emotional and cognitive outcomes for domestic violence survivors: The impact of shelter stay and self-compassion support groups. Journal Interpers Violence.

[B5-behavsci-15-00769] Altuna B. (2018). Empatía y moralidad: Las dimensiones psicológicas y filosóficas de una relación compleja. Revista de Filosofía.

[B6-behavsci-15-00769] Alzugaray C., Basabe N., Muratori M., García F., Mateos-Pérez E. (2018). Psicología comunitaria positiva y resiliencia comunitaria: Una propuesta de instrumento. Revista Latinoamericana de Psicología Positiva.

[B7-behavsci-15-00769] Alzugaray C., Mateos-Pérez E., Teletxea S. (2020). Resiliencia comunitaria y bienestar en adolescentes en desprotección socio-familiar. Inclusão Social.

[B8-behavsci-15-00769] Atkinson P. A., Martin C. R., Rankin J. (2009). Resilience revisited. Journal Psychiatric Mental Health in Nurse.

[B9-behavsci-15-00769] Banerjee Y., Akhras A., Khamis A. H., Alsheikh-Ali A., Davis D. (2019). Investigating the relationship between resilience, stress-coping strategies, and learning approaches to predict academic performance in undergraduate medical students: Protocol for a proof-of-concept study. JMIR Research Protocols.

[B10-behavsci-15-00769] Barrera-Gil D., Estrada-Méndez N., Arévalo Y., Calzadilla-Núñez A., Díaz-Narváez V. P. (2018). Empatía en estudiantes de medicina de la República de El Salvador: Estudio transversal [Empathy in medical students in the Republic of El Salvador: Cross-sectional study]. Journal of Healthcare Quality Research.

[B11-behavsci-15-00769] Berzenski S. R., Yates T. M. (2022). The development of empathy in child maltreatment contexts. Child Abuse & Neglect.

[B12-behavsci-15-00769] Blair R. J. R. (2018). Traits of empathy and anger: Implications for psychopathy and other disorders associated with aggression. Philosophical Transactions of the Royal Society B Bilogical Sciences.

[B13-behavsci-15-00769] Bonanno G. A. (2021). The resilience paradox. European Journal of Psychotraumatology.

[B14-behavsci-15-00769] Cao X., Chen L. (2020). The impact of empathy on work engagement in hemodialysis nurses: The mediating role of resilience. Japan Journal of Nursing Science.

[B15-behavsci-15-00769] Chaukos D., Chad-Friedman E., Mehta D. H., Byerly L., Celik A., McCoy T. H., Denninger J. W. (2017). Risk and resilience factors associated with resident burnout. Academic Psychiatry.

[B16-behavsci-15-00769] Cheung G. W., Rensvold R. B. (2002). Evaluating goodness-of-fit indexes for testing measurement invariance. Structural Equation Modeling: A Multidisciplinary Journal.

[B17-behavsci-15-00769] Connor K. M., Davidson J. R. (2003). Development of a new resilience scale: The Connor-Davidson resilience scale (CD-RISC). Depression and Anxiety.

[B18-behavsci-15-00769] Couette M., Mouchabac S., Adrien V., Cagnone V., Bourla A., Ferreri F. (2022). Functional neuro-anatomy of social cognition in posttraumatic stress disorder: A systematic review. Psychiatry Research.

[B19-behavsci-15-00769] Cruz K. A. (2020). Empatía en personal de salud de urgencias. Psic-Obesidad.

[B20-behavsci-15-00769] Dávila-Pontón Y., Reyes-Reyes A., Calzadilla-Núñez A., Utsman R., Torres-Martínez P. A., Díaz-Narváez V. P. (2020a). Empathy and personality styles in medical students. Revista Colombiana de Psicología.

[B21-behavsci-15-00769] Dávila-Pontón Y., Velez-Calvo X., Gomez-Coello A., Muñoz J., Aguilera, Diaz-Narváez V., Calzadilla-Núñez A., Reyes-Reyes A., Torres-Martínez P. (2020b). Empathy and family functioning in medical students of the University of Azuay, Cuenca, Ecuador. Revista Salud Uninorte.

[B22-behavsci-15-00769] Decety J., Jackson P. H. (2004). The functional architecture of human empathy. Behavioural and Cognitive Neuroscience Review.

[B23-behavsci-15-00769] Decety J., Svetlova M. (2012). Putting together phylogenetic and ontogenetic perspectives on empathy. Developmental Cognitive Neuroscience.

[B24-behavsci-15-00769] de Roover K., Vermunt J. K., Ceulemans E. (2022). Mixture multigroup factor analysis for unraveling factor loading noninvariance across many groups. Psychological Methods.

[B25-behavsci-15-00769] Díaz-Narváez V. P., Calzadilla-Núñez A., López-Orellana P., Utsman-Abarca R., Alonso-Palacio L. M. (2020). Empathic decline and training in nursing students. Revista da Escola de Enfermagem da USP.

[B26-behavsci-15-00769] Díaz-Narváez V. P., Miranda-Carreño F., Galaz-Guajardo S., Sepúlveda-Navarro W., Zúñiga-Mogollones M., Calzadilla-Núñez A., Torres-Martínez P., Reyes-Reyes A. (2021). Variability of empathy among dental students. Implications not yet understood in Latin America. Revista de la Facultad de Medicina.

[B27-behavsci-15-00769] Díaz-Narváez V. P., Silva-Vetri M. G., Stocklin B., González-Díaz E., Calzadilla-Núñez A., Torres-Martínez P., Reyes-Reyes A. (2022). Empatía en estudiantes y profesores de una escuela de odontología de República Dominicana. Revista Facultad de Medicina.

[B28-behavsci-15-00769] Dulin A. J., Dale S. K., Earnshaw V. A., Fava J. L., Mugavero M. J., Napravnik S., Hogan J. W., Carey M. P., Howe C. J. (2018). Resilience and HIV: A review of the definition and study of resilience. AIDS Care.

[B29-behavsci-15-00769] Eisenberg N., Fabes R. A. (1990). Empathy: Conceptualization, measurement, and relation to prosocial behavior. Motivation and Emotion.

[B30-behavsci-15-00769] Eisenberg N., Shea C. L., Carlo G., Knight G. P., Kurtines W. M., Gewirtz J. L. (1991). Empathy related responding and cognition: A «chicken and the egg» dilemma. Handbook of moral behavior and development.

[B31-behavsci-15-00769] Elam T., Taku K. (2022). Differences between posttraumatic growth and resiliency: Their distinctive relationships with empathy and emotion recognition ability. Frontier in Psychology.

[B32-behavsci-15-00769] Faye C., Mcgowan J. C., Denny C. A., David D. J. (2018). Neurobiological mechanisms of stress resilience and implications for the aged population. Current Neuropharmacology.

[B33-behavsci-15-00769] Férriz L., Sobral J., Gómez-Fraguela J. A. (2018). Empatía y delincuencia juvenil: Un meta-análisis sobre la relación. Revista Iberoamericana de Psicología y Salud.

[B34-behavsci-15-00769] Findyartini A., Greviana N., Putera A. M., Sutanto R. L., Saki V. Y., Felaza E. (2021). The relationships between resilience and student personal factors in an undergraduate medical program. BMC Medical Education.

[B35-behavsci-15-00769] Fonseca-Molina J., Torres-Martínez P. A., Barrios-Penna C. A., Calbacho-Contreras V., Aguirre-Bustamante J. P., Fernández-Sagredo M., Díaz-Narváez V. P. (2018a). A longitudinal study on stress sources perceived by Chilean dental students. Revista Facultad de Medicina.

[B36-behavsci-15-00769] Fonseca-Molina J., Torres-Martínez P. A., Barrios-Penna C. A., Fernández-Sagredo M., Díaz-Narváez V. P. (2018b). Perception of environment stressors in Chilean dentistry students. Pesquisa Brasileira em Odontopediatria e Clinica Integrada.

[B37-behavsci-15-00769] Foster K., Cuzzillo C., Furness T. (2018). Strengthening mental health nurses’ resilience through a workplace resilience programme: A qualitative inquiry. Journal of Psychiatric & Mental Health Nursing.

[B38-behavsci-15-00769] Ganesan K., Steinbeis N. (2022). Development and plasticity of executive functions: A value-based account. Current Opinion in Psychology.

[B39-behavsci-15-00769] Garandeau C. F., Laninga-Wijnen L., Salmivalli C. (2022). Effects of the KiVa anti-bullying program on affective and cognitive empathy in children and adolescents. Journal Clinical Child & Adolescent Psychology.

[B40-behavsci-15-00769] Gariépy G., Honkaniemi H., Quesnel-Vallée A. (2016). Social support and protection from depression: Systematic review of current findings in western countries. British Journal of Psychiatry.

[B41-behavsci-15-00769] Golubovich J., Chang C. G., Eatough E. M. (2014). Safety climate, hardiness, and musculoskeletal complaints: A mediated moderation model. Applied Ergonomics.

[B42-behavsci-15-00769] Halimi S. N., Mirzaei A., Rowett D., Whitfield K., Luetsch K. (2023). Resilience and empathy in pharmacy interns: Insights from a three-year cohort study. Exploratory Research in Clinical and Social Pharmacy.

[B43-behavsci-15-00769] Han M.-H., Nestler E. J. (2017). Neural substrates of depression and resilience. Neurotherapeutics.

[B44-behavsci-15-00769] Hartman C., Hageman T., Williams J. H., Mary J. S., Ascione F. R. (2019). Exploring empathy and callous-unemotional traits as predictors of animal abuse perpetrated by children exposed to intimate partner violence. Journal Interpers Violence.

[B45-behavsci-15-00769] Heritage B., Rees C. S., Osseiran-Moisson R., Chamberlain D., Cusack L., Anderson J., Fagence A., Sutton K., Brown J., Terry V. R., Hemsworth D., Hegney D. G. (2019). A re-examination of the individual differences approach that explains occupational resilience and psychological adjustment among nurses. Journal of Nurses Management.

[B46-behavsci-15-00769] Hoffman R. R., Hancock P. A. (2017). Measuring resilience. Human Factors.

[B47-behavsci-15-00769] Hojat M., Gonnella J. S., Mangione S., Nasca T. J., Veloski J. J., Erdmann J. B., Callahan C. A., Magee M. (2002a). Empathy in medical students as related to academic performance, clinical competence, and gender. Medical Education.

[B48-behavsci-15-00769] Hojat M., Gonnella J. S., Nasca T. J., Mangione S., Veloski J. J., Magee M. (2002b). The Jefferson scale of physician empathy: Further psychometric data and differences by gender and specialty at item level. Academic Medicine.

[B49-behavsci-15-00769] Hojat M., Maio V., Pohl C. A., Gonella J. S. (2023). Clinical empathy: Definition, measurement, correlates, group differences, erosion, enhancement, and healthcare outcomes. Discover Health Systems.

[B50-behavsci-15-00769] Hojat M., Shannon S. C., De Santis J., Speicher M. R., Bragan L., Calabrese L. H. (2020). Does empathy decline in the clinical phase of medical education? A nationwide, multi-institutional, cross-sectional study of students at DO-granting medical schools. Academic Medicine.

[B51-behavsci-15-00769] Holling C. S., Schulze P. (2006). Engineering resilience versus ecological resilience. Engineering within ecological constraints.

[B52-behavsci-15-00769] Howell K. H., Miller-Graff L. E. (2014). Protective factors associated with resilient functioning in young adulthood after childhood exposure to violence. Child Abuse and Neglect.

[B53-behavsci-15-00769] Hoyle R. H., Hoyle R. H. (1995). The structural equation modeling approach: Basic concepts and fundament al issues. Structural equation modeling: Concepts, issues, and applications.

[B54-behavsci-15-00769] Hu L. T., Bentler P. M. (1999). Cutoff criteria for fit indexes in covariance structure analysis: Conventional criteria versus new alternatives. Structural Equation Modeling.

[B55-behavsci-15-00769] Hunsu N. J., Oje A. V., Tanner-Smith E. E., Adesope O. (2023). Relationships between risk factors, protective factors and achievement outcomes in academic resilience research: A meta-analytic review. Educational Research Review.

[B56-behavsci-15-00769] Johnson J., Wood A. M., Gooding P., Tarrier N. (2011). Resilience to suicidality: The buffering hypothesis. Clinical Psychology Review.

[B57-behavsci-15-00769] Jöreskog K. G. (1971b). Simultaneous factor analysis in several populations. Psychometrika.

[B58-behavsci-15-00769] Kerr-Gaffney J., Harrison A., Tchanturia K. (2019). Cognitive and affective empathy in eating disorders: A systematic review and meta-analysis. Frontiers in Psychiatry.

[B59-behavsci-15-00769] Khalil M., Ravaghi H., Samhouri D., Abo J., Ali A., Sakr H., Camacho A. (2022). What is “hospital resilience”? A scoping review on conceptualization, operationalization, and evaluation. Frontiers in Public Health.

[B60-behavsci-15-00769] Kline R. B. (2005). Principles and practice of structural equation modeling.

[B61-behavsci-15-00769] Lopes A. R., Nihei O. K. (2020). Burnout among nursing students: Predictors and association with empathy and self-efficacy. Revista Brasileira de Enfermagem.

[B62-behavsci-15-00769] MacAulay R., Morash J., Kenwell L. S., Haslam S. K. (2023). Burnout in oral health students: A scoping review. Journal of Dental Education.

[B63-behavsci-15-00769] Machado S., Calvetti P. (2019). Instrumentos psicométricos para avaliação da empatia em crianças e adolescentes: Revisão sistemática. Psicologia em Revista.

[B64-behavsci-15-00769] Maltby J., Day L., Hall S. (2015). Refining trait resilience: Identifying engineering, ecological, and adaptive facets from extant measures of resilience. PLoS ONE.

[B65-behavsci-15-00769] Maltby J., Day L., Zemojtel-Piotrowska M., Piotrowski J., Hitokoto H., Baran T., Flowe H. D. (2016). An ecological systems model of trait resilience: Cross-cultural and clinical relevance. Personality and Individual Differences.

[B66-behavsci-15-00769] Metcalf S., Dickerson K. L., Milojevich H. M., Quas J. A. (2021). Primary and secondary variants of psychopathic traits in at-risk youth: Links with maltreatment, aggression, and empathy. Child Psychiatry & Human Development.

[B67-behavsci-15-00769] Morice-Ramat A., Goronflot L., Guihard G. (2018). Are alexithymia and empathy predicting factors of the resilience of medical residents in France?. International Journal Medical Education.

[B68-behavsci-15-00769] Moya J., Goenechea M. (2022). An approach to the unified conceptualization, definition, and characterization of social resilience. International Journal of Environmental Research and Public Health.

[B69-behavsci-15-00769] Nitschke J. P., Bartz J. A. (2023). The association between acute stress & empathy: A systematic literature review. Neuroscience Biobehavioral Reviews.

[B70-behavsci-15-00769] Ord A. S., Stranahan K. R., Hurley R. A., Taber K. H. (2020). Stress-related growth: Building a more resilient brain. Journal of Neuropsychiatry & Clinical Neurosciences.

[B71-behavsci-15-00769] Palacio G. C., Krikorian A., Gómez-Romero M. J., Limonero J. T. (2020). Resilience in caregivers: A systematic review. American Journal of Hospice and Palliative Care.

[B72-behavsci-15-00769] Paloniemi E., Mikkola I., Vatjus R., Jokelainen J., Timonen M., Hagnäs M. (2021). Measures of empathy and the capacity for self-reflection in dental and medical students. BMC Medical Education.

[B73-behavsci-15-00769] Rafaqat W., Sami A., Ibrahim M. T., Ibad H., Awais S., Memon A., Shahbaz F. F., Ahmed D., Zindani S., Leghari A. L., Saleem S. (2022). Impact of perfectionism and resilience on empathy in medical students: A cross-sectional study. Journal of Patient Experience.

[B74-behavsci-15-00769] Rajput S., Puranik M. P., Shanbhag N., Kumar A. (2020). Factors affecting empathy among Indian dentists. Indian Journal of Dental Research.

[B75-behavsci-15-00769] Ramos M. C., Miller K. F., Moss I. K., Margolin G. (2021). Perspective-taking and empathy mitigate family-of-origin risk for electronic aggression perpetration toward dating partners: A brief report. Journal of Interpersonal Violence.

[B76-behavsci-15-00769] Savin N. E., White K. J. (1977). The Durbin-Watson test for serial correlation with extreme sample sizes or many regressors. Econometrica.

[B77-behavsci-15-00769] Shibaoka M., Masuda M., Iwasawa S., Ikezawa S., Eguchi H., Nakagome K. (2023). Relationship between objective cognitive functioning and work performance among Japanese workers. Journal of Occupational Health.

[B78-behavsci-15-00769] Singh R., Mahato S., Singh B., Thapa J., Gartland D. (2019). Resilience in Nepalese adolescents: Socio-demographic factors associated with low resilience. Journal of Multidisciplinary Healthcare.

[B79-behavsci-15-00769] Sisto A., Vicinanza F., Campanozzi L. L., Ricci G., Tartaglini D., Tambone V. (2019). Towards a transversal definition of psychological resilience: A literature review. Medicina.

[B80-behavsci-15-00769] Spilg E. G., McNeill K., Sabri E., Duffy M. C., Ananny L., Graham I. D., LeBlanc V., Wells P. S. (2022). A cross-sectional study of the interrelationship between burnout, empathy and resilience in academic physicians. Psychology, Health & Medicine.

[B81-behavsci-15-00769] Staneva A., Carmignani F., Rohde N. (2022). Personality, gender, and age resilience to the mental health effects of COVID-19. Social Science & Medicine.

[B82-behavsci-15-00769] Stern Y., Albert M., Barnes C. A., Cabeza R., Pascual-Leone A., Rapp P. R. (2023). A framework for concepts of reserve and resilience in aging. Neurobiology of Aging.

[B83-behavsci-15-00769] Sturzu L., Lala A., Bisch M., Guitter M., Dobre D., Schwan R. (2019). Empathy and burnout—A cross-sectional study among mental healthcare providers in France. Journal of Medicine and Life.

[B84-behavsci-15-00769] Suazo I., Pérez-Fuentes M. D. C., Molero Jurado M. D. M., Martos Martínez Á., Simón Márquez M. D. M., Barragán, Martín A. B., Sisto M., Gázquez Linares J. J. (2020). Moral sensitivity, empathy and prosocial behavior: Implications for humanization of nursing care. International Journal of Environmental Research and Public Health.

[B85-behavsci-15-00769] Tang Y., He C., Feng L., Wu D., Zhou X., Li T., He L., Cai Q., Yue Y. (2022). The impact of implicit theories on resilience among Chinese nurses: The chain mediating effect of grit and meaning in life. Frontier in Psychology.

[B86-behavsci-15-00769] Tehranineshat B., Rakhshan M., Torabizadeh C., Fararouei M. (2019). Compassionate care in healthcare systems: A systematic review. Journal of the National Medical Association.

[B87-behavsci-15-00769] Thompson N. M., Uusberg A., Gross J. J., Chakrabarti B. (2019). Empathy and emotion regulation: An integrative account. Progress in Brain Research.

[B88-behavsci-15-00769] Timpane-Padgham B. L., Beechie T., Klinger T. (2017). A systematic review of ecological attributes that confer resilience to climate change in environmental restoration. PLoS ONE.

[B89-behavsci-15-00769] Tokgöz Kaplan T. (2024). Stress levels amongst Turkish dental students, general dentists and paediatric dentists during performing paediatric dentistry: A questionnaire-based cross-sectional study. European Archives of Paediatric Dentistry.

[B90-behavsci-15-00769] Ueno Y., Hirano M., Oshio A. (2021). Resilience and socio-demographic characteristics: Results from a large cross-sectional study in Japan. Originals.

[B91-behavsci-15-00769] Ulloque M. J., Villalba S., Varela de Villalba T., Fantini A., Quinteros S., Díaz-Narváez V. (2019). Empathy in medical students of Córdoba, Argentina. Archivos Argentinos de Pediatría.

[B92-behavsci-15-00769] Ullrich H. E. (2022). Culture, empathy, and the therapeutic alliance. Psychodynamic Psychiatry.

[B93-behavsci-15-00769] Vaughn L. M., DeJonckheere M. (2021). The opportunity of social ecological resilience in the promotion of youth health and wellbeing: A narrative review. Yale Journal of Biology and Medicine.

[B94-behavsci-15-00769] Velayudhan B. V. (2021). Embracing uncertainty: An empathy and resilience-based approach to cardiothoracic surgery in a post-pandemic era. Indian Journal of Thoracic and Cardiovascular Surgery.

[B95-behavsci-15-00769] Velickovic K., Rahm, Hallberg I., Axelsson U., Borrebaeck C. A. K., Rydén L., Johnsson P., Månsson J. (2020). Psychometric properties of the Connor-Davidson Resilience Scale (CD-RISC) in a non-clinical population in Sweden. Health and Quality of Life Outcomes.

[B96-behavsci-15-00769] Waddimba A. C., Bennett M. M., Fresnedo M., Ledbetter T. G., Warren A. M. (2021). Resilience, well-being, and empathy among private practice physicians and advanced practice providers in Texas: A structural equation model study. Mayo Clinic Proceedings: Innovations, Quality & Outcomes.

[B97-behavsci-15-00769] Wu W., Ma X., Liu Y., Qi Q., Guo Z., Li S., Yu L., Long Q., Chen Y., Teng Z., Li X., Zeng Y. (2022). Empathy alleviates the learning burnout of medical college students through enhancing resilience. BMC Medical Education.

[B98-behavsci-15-00769] Young C., Roberts R., Ward L. (2019). Application of resilience theories in the transition to parenthood: A scoping review. Journal of Reproductive and Infant Psychology.

[B99-behavsci-15-00769] Yule K., Houston J., Grych J. (2019). Resilience in children exposed to violence: A meta-analysis of protective factors across ecological contexts. Clinical Child and Family Psychology Review.

[B100-behavsci-15-00769] Zhang J., Wang X., Xu T., Li J., Li H., Wu Y., Li Y., Chen Y., Zhang J. P. (2022). The effect of resilience and self-efficacy on nurses’ compassion fatigue: A cross-sectional study. Journal of Advanced Nursing.

